# Exploratory Metabolomic Analyses Reveal Compounds Correlated with Lutein Concentration in Frontal Cortex, Hippocampus, and Occipital Cortex of Human Infant Brain

**DOI:** 10.1371/journal.pone.0136904

**Published:** 2015-08-28

**Authors:** Jacqueline C. Lieblein-Boff, Elizabeth J. Johnson, Adam D. Kennedy, Chron-Si Lai, Matthew J. Kuchan

**Affiliations:** 1 Research and Development, Abbott Nutrition, Columbus, Ohio, United States of America; 2 Antioxidants Research Laboratory, Jean Mayer United States Department of Agriculture Human Nutrition Research Center on Aging, Tufts University, Boston, Massachusetts, United States of America; 3 Metabolon, Incorporated, Durham, North Carolina, United States of America; Zhejiang Key Laborotory for Research in Assesment of Cognitive Impairments, CHINA

## Abstract

Lutein is a dietary carotenoid well known for its role as an antioxidant in the macula, and recent reports implicate a role for lutein in cognitive function. Lutein is the dominant carotenoid in both pediatric and geriatric brain tissue. In addition, cognitive function in older adults correlated with macular and postmortem brain lutein concentrations. Furthermore, lutein was found to preferentially accumulate in the infant brain in comparison to other carotenoids that are predominant in diet. While lutein is consistently related to cognitive function, the mechanisms by which lutein may influence cognition are not clear. In an effort to identify potential mechanisms through which lutein might influence neurodevelopment, an exploratory study relating metabolite signatures and lutein was completed. Post-mortem metabolomic analyses were performed on human infant brain tissues in three regions important for learning and memory: the frontal cortex, hippocampus, and occipital cortex. Metabolomic profiles were compared to lutein concentration, and correlations were identified and reported here. A total of 1276 correlations were carried out across all brain regions. Of 427 metabolites analyzed, 257 were metabolites of known identity. Unidentified metabolite correlations (510) were excluded. In addition, moderate correlations with xenobiotic relationships (2) or those driven by single outliers (3) were excluded from further study. Lutein concentrations correlated with lipid pathway metabolites, energy pathway metabolites, brain osmolytes, amino acid neurotransmitters, and the antioxidant homocarnosine. These correlations were often brain region—specific. Revealing relationships between lutein and metabolic pathways may help identify potential candidates on which to complete further analyses and may shed light on important roles of lutein in the human brain during development.

## Introduction

Lutein is a naturally occurring xanthophyll carotenoid found in fruits and vegetables, with green leafy vegetables such as spinach and kale accounting for the most abundant sources in nature [1]. Animals cannot synthesize lutein, and therefore, it can only be acquired in the body through diet. Lutein and zeaxanthin, a closely associated carotenoid, are the only carotenoids that constitute the yellow pigment characteristic of the macula [2]. These carotenoids are important for absorbing high energy blue light and protecting the photoreceptor cell layer from oxidative damage [3–5]. Lutein has also been implicated in protective roles for other tissues in the body. For instance, epidemiological and preclinical studies suggest that lutein intake may moderate progression of early atherosclerosis [6]. Lutein may also help protect the skin against the harmful oxidative effects of UV exposure [7, 8]. In addition, a high carotenoid diet, including lutein, may reduce the risk of some types of cancer [9].

Recently, serum and brain levels of lutein were reported to be positively associated with improved cognitive function in the elderly [10], and supplementation of older women with lutein improved cognitive scores after 4 months [11]. Cognition scores in older adults with mild cognitive impairment correlated with macular pigment optical density, a measure of the concentration of lutein and zeaxanthin in the macula [12–14]. Lutein is one of the prevalent carotenoids in mature breast milk [15, 16] and was recently reported to be the predominant carotenoid in the developing infant brain [17]. A recent clinical trial demonstrated that lutein supplementation may improve neuroretinal health in preterm newborn infants [18]. Taken together, these findings suggest that lutein may be important for cognition in the elderly and for neurodevelopment in the infant. However, the mechanisms by which lutein may influence these processes are largely not understood.

Metabolomics, the analysis of small molecule products of cellular metabolism (e.g., sugars, organic acids, amino acids, and nucleotides), is a modern technique often used for studying the complex impact of nutrients on biological tissues [19–22]. By analyzing the full metabolite complement of a cell, tissue, or organism, metabolomics can help reveal the interaction of nutrients with metabolite pathways. The metabolome is context-dependent and can change in response to external factors, including nutrient intake and availability. Thus, measuring the changes in the amount and identity of metabolites in relation to tissue concentrations of lutein can help provide insight into the biochemistry underlying the response to lutein.

Here, we conducted exploratory metabolomic analyses of postmortem infant brain samples to establish testable hypotheses that might explain the importance of lutein in brain development. We found that lutein concentrations correlated with lipid pathway metabolites, energy pathway metabolites, brain osmolytes, amino acid neurotransmitters, and the antioxidant homocarnosine, and these correlations were often in a brain region specific manner.

## Methods

### Subjects

Brain samples were obtained with permission from the National Institute of Child Health and Human Development Brain and Tissue Bank for Developmental Disorders at the University of Maryland. The collection protocol used at the University of Maryland, Baltimore to obtain decedent tissues was reviewed and approved by the Institutional Review Board of the University of Maryland, Baltimore and by the Institutional Review Board of the Maryland Department of Health and Mental Hygiene. Analyses were performed on de-identified brain tissue. Per federal guidance, this activity does not constitute human subject research. Tissues were from otherwise healthy infants (without any brain and/or other systemic pathologies) and were voluntarily donated and distinguished using a unique numerical identifier which obscured the identity of the decedent. Information about decedent characteristics is available in Table 1. Thirty total decedents were studied and included both male (n = 21) and female (n = 9) infants. A total of 81 tissues were analyzed from three regions of the brain commonly analyzed for learning and memory, including the frontal cortex, a region involved in executive function (n = 29), hippocampus, a region involved in memory (n = 24), and the occipital cortex, a region involved in vision (n = 28). Twenty-two of the thirty decedents had tissues from all three regions of the brain. Because of the limited quantity of tissues available from the brain bank, frontal cortex was unavailable for one decedent, hippocampus was unavailable for five decedents, occipital cortex was unavailable for one decedent, and both hippocampus and occipital cortex were unavailable for one decedent. Tissues were stored frozen (-70°C) until analyses.

**Table 1:** Decedent Characteristics.

**Age, days**	
Mean (SEM)	137 (21)
Median	100
Range	1–488
**Sex distribution**	
Males, n (%)	21 (70.0%)
**Race**	
African American, n (%)	12 (40.0%)
Caucasian, n (%)	16 (53.3%)
Hispanic, n (%)	2 (6.7%)
**Cause of death**	
SIDS, n (%)	15 (50.0%)
Others, n (%)	15 (50.0%)
Asphyxia	1
Asthma	1
Bronchopneumonia	4
Cardiac Arrhythmia	1
Congenital Heart Defects	2
Complications of prematurity, bronchopulmonary dysplasia	1
Dehydration	1
Drowning	1
Hyperthermia	1
Multiorgan failure	1
Pneumonia associated with meconium aspiration	1
**Time interval between death and tissue collection, hours**	
Mean (SEM)	15.9 (1.1)
Median	17.5
Range	2–23

### Lutein Concentrations in Infant Brain

Lutein concentration analysis in these brain tissues was previously described and reported [17]. Carotenoids were extracted from brain tissue from each region by homogenizing in an ethanol-saline solution. An internal standard (echinenone) was added and incubated in a 70°C water bath for 2 minutes. A sodium ascorbate (25%) and sodium hydroxide (5%) solution was added and incubated in a 60°C water bath for 20 minutes. Distilled water was added and the solution was cooled for 5 minutes. Hexane was added to the solution and vortexed vigorously prior to centrifugation at 1000 x g for 10 minutes at 4°C. The upper hexane layer was removed and evaporated under nitrogen in a 40°C water bath. Extraction was repeated and both hexane layers combined. Evaporation continued until dryness, and vessel rinsed with hexane and evaporation completed again. The dried residue was reconstituted with 100 μl of a 1:1 mixture of ethanol and methyl tert butyl ester, vortexed, sonicated, and transferred to HPLC inserts and centrifuged at 2000 x g for 3 minutes to remove precipitate. The clear supernatant was transferred to a clean insert. Extracts were analyzed using reverse-phase HPLC using a method described by Yeum et al. with a C30 carotenoid column (3 μm, 150 x 4.6 mm, YMC, Wilmington, NC) [23]. Data were expressed per wet weight of tissue.

### Metabolomic Analysis

All brain tissue samples were processed by Metabolon, Inc. (Research Triangle Park, NC) using their standard extraction protocol [24]. Starting with 100 mg of tissue, small molecules were extracted in an 80% methanol solution containing four standards (tridecanoic acid, 4-Cl-phenylalanine, 2-flurophenylglycine, and d6-cholesterol) used to monitor extraction efficiency. After metabolite extraction from each sample, clarified supernatants were split into three equal parts and dried under nitrogen gas for analyses on gas chromatography/mass spectrometry (GC/MS) and liquid chromatography/mass spectrometry (LC/MS) platforms. For one aliquot, analytes were derivatized using bistrimethyl-silyl-trifluoroacetamide and analyzed on a Trace DSQ fast-scanning single-quadruple mass spectrometer (Thermo-Finnigan). For the remaining two aliquots, one specimen was resuspended in 50 μl of 6.5 mM ammonium bicarbonate, pH 8, for liquid chromatography mass spectrometry (LC/MS) analysis in negative ion mode; and the other was resuspended in 50 μl of 0.1% formic acid in 10% methanol for LC/MS analysis in positive ion mode. Both resuspension buffers contained instrument internal isotopic standards used to monitor performance and serve as retention index markers. Standards for negative ion mode analyses included d7-glucose, d3-methionine, d3-leucine, d8-phenylalanine, d5-tryptophan, Cl-phenylalanine, Br-phenylalanine, d15-octanoic acid, d19-decanoic acid, d27-tetradecanoic acid, and d35-octadecanoic acid. Standards for positive ion mode analyses included d7-glucose, fluorophenylglycine, d3-methionine, d4-tyrosine, d3-leucine, d8-phenylalanine, d5-tryptophan, d5-hippuric acid, Cl-phenylalanine, Br-phenylalanine, d5-indole acetate, d9-progesterone, and d4-dioctylpthalate. Internal standards were chosen based on their broad chemical structures, biological variety and their elution spectrum on each of the arms of the platform. Chromatographic separation was completed using an ACQUITY UPLC (Waters) equipped with a Waters BEH C18 column followed by analysis with an LTQ mass spectrometer (Thermo-Finnigan) [24]. Following chromatographic separation, full-scan mass spectroscopy was applied to record and quantify all detectable ions present in samples. Metabolites with known chemical structures were identified by matching the chromatographic retention index for each ion and mass spectral fragmentation signatures with reference library entries. For ions not covered by these standards, additional library entries were established from their unique ion signatures. To monitor process variability, the median relative standard deviation (RSD) was calculated for all spiked standards (listed above) using median scaled values. Overall, spiked extraction standards and instrument internal standards detected a median process variability of ≤5%. Metabolite intensities were median-scaled for each biochemical, and qualitative levels in intensity were reported. These scaled intensity values were then compared to lutein concentrations independently determined by HPLC (previously reported, [17]) for the same subject samples.

### Statistical Analysis

To test for correlations between lutein concentrations and age, data were analyzed using Pearson’s correlation procedures of GraphPad Prism version 5.04 for Windows, GraphPad Software, La Jolla California USA, www.graphpad.com. To test for correlations between lutein concentrations and metabolites, data were analyzed using Pearson’s correlation procedures of Array Studio Software, OmicSoft Corporation, Cary, NC, USA http://www.omicsoft.com/array-studio/. All metabolites with values producing a moderate (r ≥ |0.45| and P < 0.05) or strong (r ≥ |0.6| and P < 0.05) correlation with lutein concentration were reported. A complete listing of all metabolite to lutein correlations can be found in the S1 Dataset. Because this project was exploratory in nature, Bonferroni corrections were not applied. Metabolites with single outliers driving correlation values were excluded from this report.

A total of 1276 correlations were carried out across all brain regions. Five metabolites fell below the detection limit in the frontal cortex, and therefore, correlations were not carried out for these. Of 427 metabolites analyzed, 257 were metabolites of known identity. Thus, 510 correlations from unidentified metabolites (170 unknown in 3 brain regions) were excluded from further analysis. In addition, 5 correlations with an r ≥ |0.45| and P < 0.05 were excluded from this report, including 3 correlations where a single outlier drove the correlation value and 2 chemical xenobiotic correlations. Using P < 0.05, it is estimated that approximately 5% of correlations were by random chance, which equates to approximately 64 comparisons out of the original 1276 correlations.

## Results

### Lutein accretion in infant brain did not correlate with age, sex, or post-mortem interval

**Fig 1:**
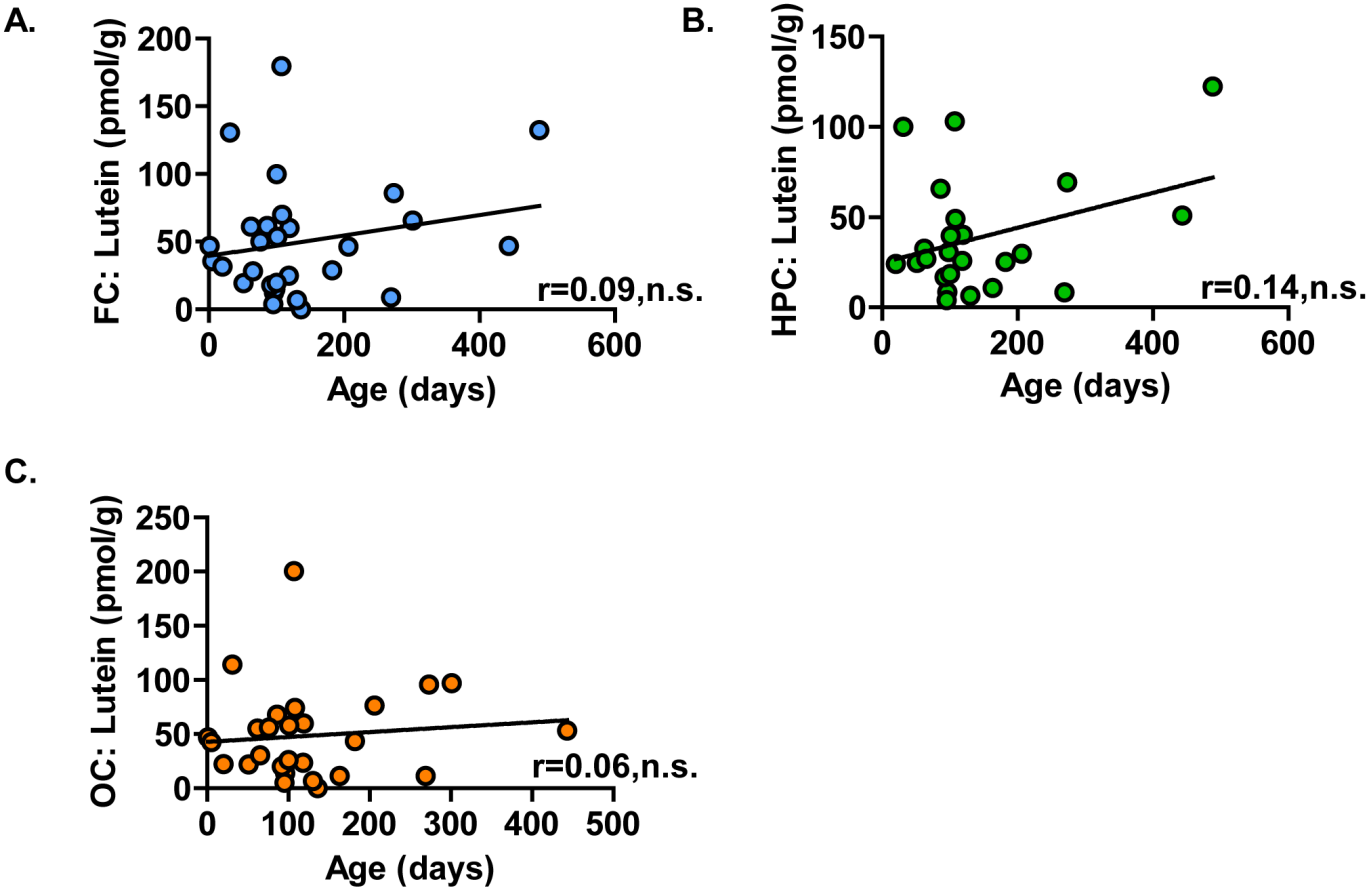
Lutein accretion in infant brain did not correlate with age. Post-mortem infant brain tissues (age 1 to 488 days) were analyzed for lutein by HPLC. Increasing lutein concentration (pmol/g) did not correlate with age in the (A) frontal cortex (n = 29), (B) hippocampus (n = 24), or (C) occipital cortex (n = 28). FC: frontal cortex; HPC: hippocampus; OC: occipital cortex; n.s.: not-significant.

The infant brain samples in this study were previously analyzed for carotenoid content, and lutein concentrations were similar across the frontal cortex, hippocampus, and occipital cortex [17]. Analyses conducted here revealed that lutein concentrations did not correlate with age in any of the three brain regions analyzed (Fig 1A–1C). In addition, lutein concentrations did not correlate with sex (S1 Fig), nor did lutein correlate with post-mortem interval, which is the time between death and tissue collection (S2 Fig). Furthermore, all metabolites were analyzed against post-mortem interval. Out of the 257 known-identity metabolites analyzed, only one produced a moderate correlation (r ≥ |0.45|) with post-mortem interval (allo-threonine, r = -0.45, P < 0.0001) (S2 Dataset).

### Fatty acids and lysophospholipids correlated with lutein concentrations in infant brain

**Fig 2:**
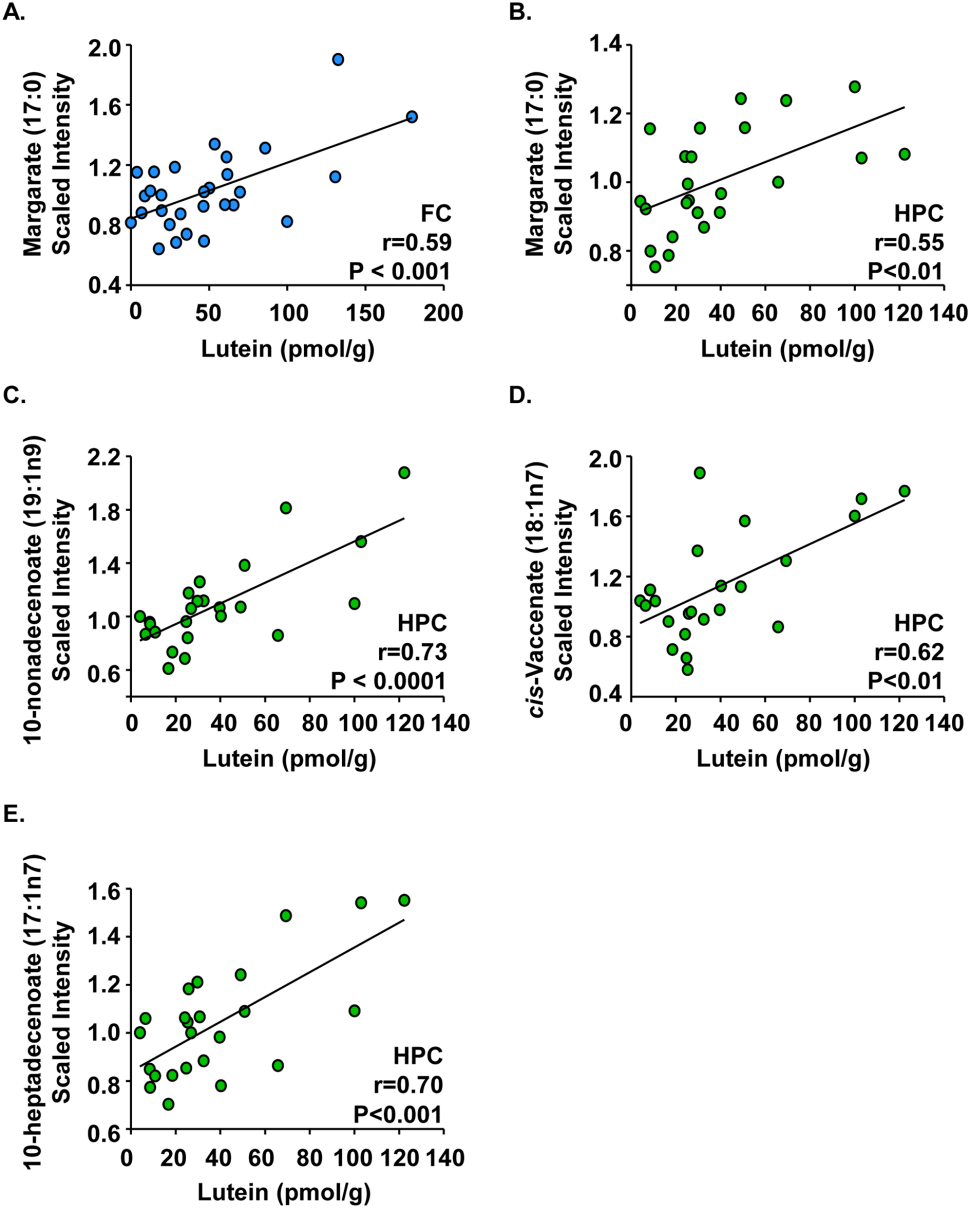
Fatty acids correlated with lutein concentrations in infant brain. Post-mortem infant brain tissues (age 1 to 488 days) from the frontal cortex, hippocampus, and occipital cortex were analyzed for both lutein and lipid pathway metabolites. Results are shown as metabolite (scaled intensity) by lutein concentration (pmol/g). In the frontal cortex (n = 29), (A) margarate had a strong, positive correlation with lutein. In the hippocampus (n = 24), (B) margarate, (C) 10-nonadecenoate, (D) *cis*-vaccenate, and (E) 10-heptadecenoate all had strong, positive correlations with lutein. No strong correlations were observed in the occipital cortex. Lipid pathway metabolite:lutein correlations with r values ≥ |0.6| and P < 0.05 are shown. FC: frontal cortex; HPC: hippocampus.

Metabolomic profiling was compared to lutein concentrations in infant frontal cortex, hippocampus, and occipital cortex. Various fatty acids correlated with lutein concentrations in these regions. Fig 2 shows that margarate positively correlated with lutein concentrations in the frontal cortex (Fig 2A, r = 0.59, P < 0.001) and the hippocampus (Fig 2B, r = 0.55, P < 0.01). Several other fatty acids also increased with lutein concentrations in the hippocampus, including 10-nonadecenoate (Fig 2C, r = 0.73, P < 0.0001), *cis*-vaccenate (Fig 2D, r = 0.62, P < 0.01), and 10-heptadecenoate (Fig 2E, r = 0.70, P < 0.001). A complete list of fatty acids that exhibited moderate to strong correlations to lutein in infant brain is provided in Table 2.

**Table 2:** Fatty acid correlations with lutein in human infant brain.^a^

Fatty acid	Correlations (r) by brain region^b^		
	FC	HPC	OC
caproate (6:0)	-0.46	-	-
pelargonate (9:0)	-	0.46	-
laurate (12:0)	0.45	-	-
palmitate (16:0)	0.49	-	-
margarate (17:0)	0.59	0.55	0.46
10-heptadecenoate (17:1n7)	0.49	0.70	-
*cis*-vaccenate (18:1n7)	0.46	0.62	-
10-nonadecenoate (19:1n9)	0.45	0.73	-

^a^Subject ages ranged from 1 to 488 days old, and the median age was 100.5 days. 30 total decedents were studied and included both male (n = 21) and female (n = 9) infants. A total of 81 tissues were analyzed from three brain regions, including the frontal cortex (FC, n = 29), hippocampus (HPC, n = 24), and the occipital cortex (OC, n = 28).

^b^All values are significant (P < 0.05). Fatty acid metabolites:lutein correlations with r values ≥ |0.45| and P < 0.05 are reported.

A wide range of lysophospholipids, intermediates of lipid metabolic pathways such as phospholipid synthesis, positively correlated with lutein concentrations in the frontal cortex, hippocampus, and occipital cortex (Table 3). Of the three brain regions studied, the frontal cortex had the strongest and greatest number of correlations between lysophospholipids and lutein. Together, these data demonstrate that numerous fatty acids and lysophosopholipids correlated with lutein concentrations in human infant brain.

**Table 3:** Lysophospholipid correlations with lutein in human infant brain.^a^

Lysophospholipid	Correlations (r) by brain region^b^		
	FC	HPC	OC
1-stearoylglycerophosphoethanolamine	-	0.52	-
1-oleoylglycerophosphoethanolamine	-	-	0.49
2-oleoylglycerophosphoethanolamine	0.48	0.46	-
1-oleoylglycerophosphocholine	-	-	0.50
2-oleoylglycerophosphocholine	0.45	-	0.46
1-palmitoylglycerophosphoinositol	0.58	-	-
1-stearoylglycerophosphoinositol	0.50	-	0.48
1-oleoylglycerophosphoinositol	0.51	-	-
1-arachidonoylglycerophosphoinositol	0.61	-	-
1-oleoylglycerophosphoserine	0.53	-	0.46
2-oleoylglycerophosphoserine	0.56	-	-

^a^Subject ages ranged from 1 to 488 days old, and the median age was 100.5 days. 30 total decedents were studied and included both male (n = 21) and female (n = 9) infants. A total of 81 tissues were analyzed from three brain regions, including the frontal cortex (FC, n = 29), hippocampus (HPC, n = 24), and the occipital cortex (OC, n = 28).

^b^All values are significant (P < 0.05). Lysophospholipid metabolites:lutein correlations with r values ≥ |0.45| and P < 0.05 are reported.

### 1-Octadecanol, Phosphate, and NADH concentrations are related to lutein concentrations in pediatric occipital cortex

**Fig 3:**
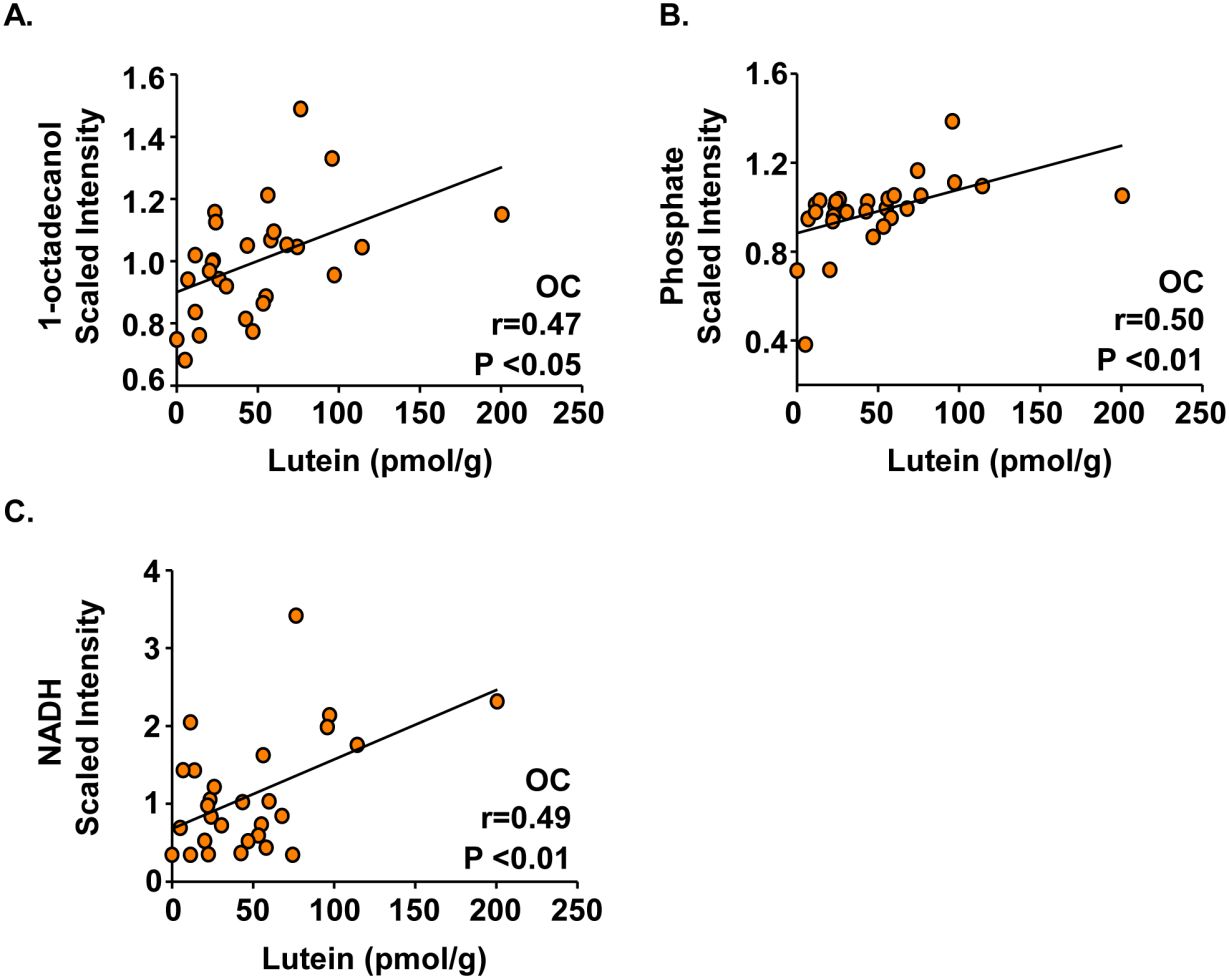
1-Octadecanol, Phosphate, and NADH concentrations are related to lutein concentrations in pediatric occipital cortex. Post-mortem infant brain tissues (age 1 to 488 days) from the frontal cortex, hippocampus, and occipital cortex were analyzed for lutein, fatty alcohol metabolites, and energy pathway metabolites. Moderate, positive correlations between lutein and (A) 1-octadecanol, (B) phosphate, and (C) NADH were unique to the occipital cortex (n = 28). Results are shown as metabolite (scaled intensity) by lutein concentration (pmol/g). Metabolite:lutein correlations with r values ≥ |0.45| and P < 0.05 are reported.

Correlations between lutein concentrations and three metabolites were observed in the occipital cortex, but not the frontal cortex or hippocampus. 1-Octadecanol, a fatty alcohol, increased with higher concentrations of lutein in the occipital cortex (Fig 3A, r = 0.47, P < 0.05). Similarly, two energy pathway metabolites, phosphate (Fig 3B, r = 0.49, P < 0.01) and NADH (Fig 3C, r = 0.50, P < 0.01), were positively correlated with lutein concentrations only in the occipital cortex.

### Taurine negatively correlated with lutein concentrations in pediatric hippocampus

**Fig 4:**
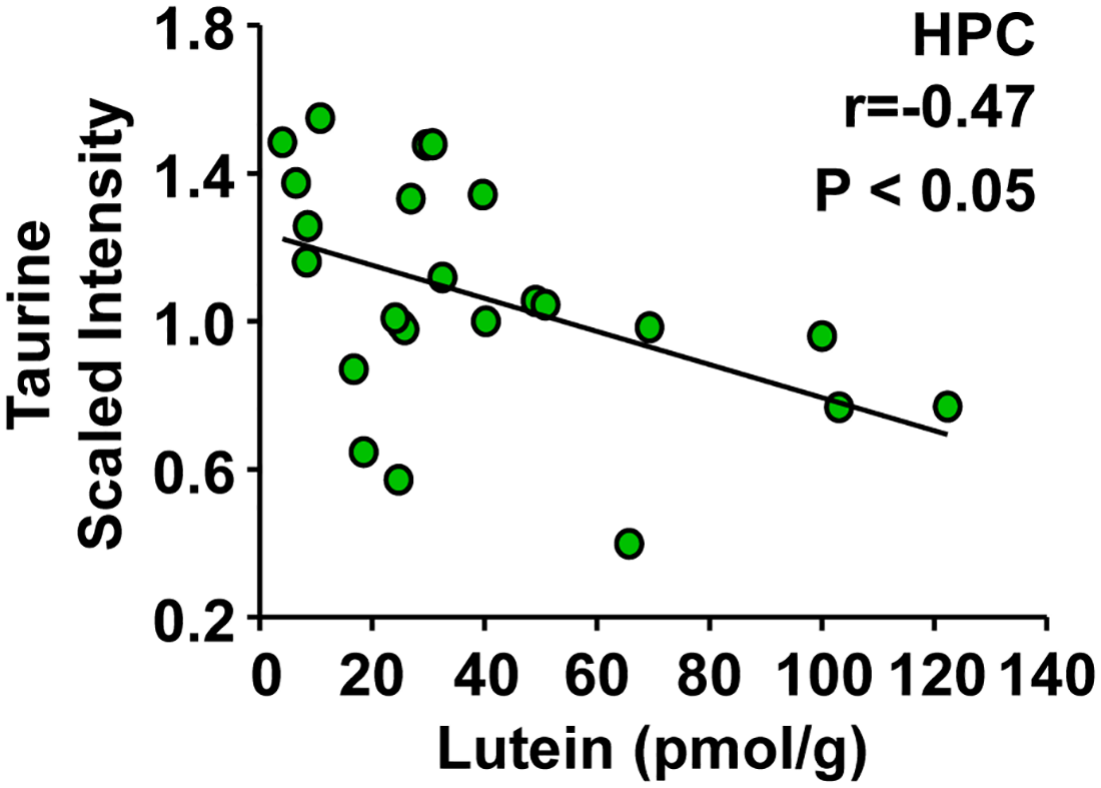
Taurine negatively correlated with lutein concentrations in pediatric hippocampus. Post-mortem infant brain tissues (age 1 to 488 days) from the frontal cortex, hippocampus, and occipital cortex were analyzed for lutein, fatty alcohol metabolites, and energy pathway metabolites. A negative correlation between taurine and lutein concentrations was unique to the hippocampus (n = 24). Results are shown as taurine (scaled intensity) by lutein concentration (pmol/g). Taurine:lutein correlations with r values ≥ |0.45| and P < 0.05 are reported.

A negative correlation was observed between lutein concentrations and taurine, a metabolite thought to be an important brain osmolyte [25] that may also act as a neurotransmitter [26–28]. This correlation was observed in the hippocampus (Fig 4, r = -0.47, P < 0.05), but not the frontal or occipital cortices.

### *scyllo*-Inositol is positively correlated with lutein concentrations in infant frontal cortex, hippocampus, and occipital cortex

**Fig 5:**
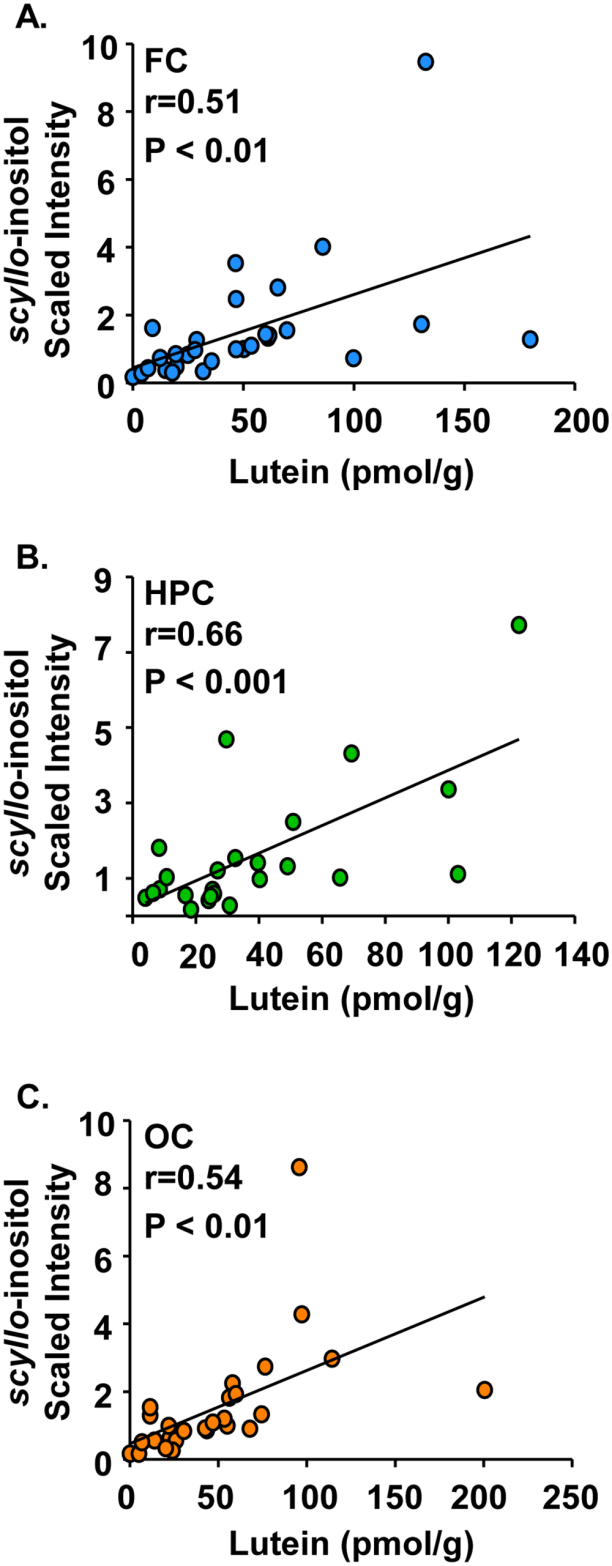
*scyllo*-Inositol is positively correlated with lutein concentrations in infant frontal cortex, hippocampus, and occipital cortex. Post-mortem infant brain tissues (age 1 to 488 days) from the frontal cortex, hippocampus, and occipital cortex were analyzed for both lutein and *scyllo*-inositol. Results are shown as *scyllo*-inositol (scaled intensity) by lutein concentration (pmol/g). Positive correlations between *scyllo*-inositol and lutein were observed in the (A) frontal cortex (n = 29), (B) hippocampus (n = 24), or (C) occipital cortex (n = 28). *scyllo*-Inositol:lutein correlations with r values ≥ |0.45| and P < 0.05 are reported. FC: frontal cortex; HPC: hippocampus; OC: occipital cortex.

*scyllo*-Inositol, an inositol isomer that may function as an osmolyte in brain [29], positively correlated with lutein in the frontal cortex (Fig 5A, r = 0.51, P < 0.01), hippocampus (Fig 5B, r = 0.66, P < 0.001), and occipital cortex (Fig 5C, r = 0.54, P < 0.01). Correlations were not observed between lutein and *myo*-inositol, the predominant inositol stereoisomer in brain.

### Amino acid neurotransmitters are positively correlated with lutein concentrations in infant hippocampus and occipital cortex

**Fig 6:**
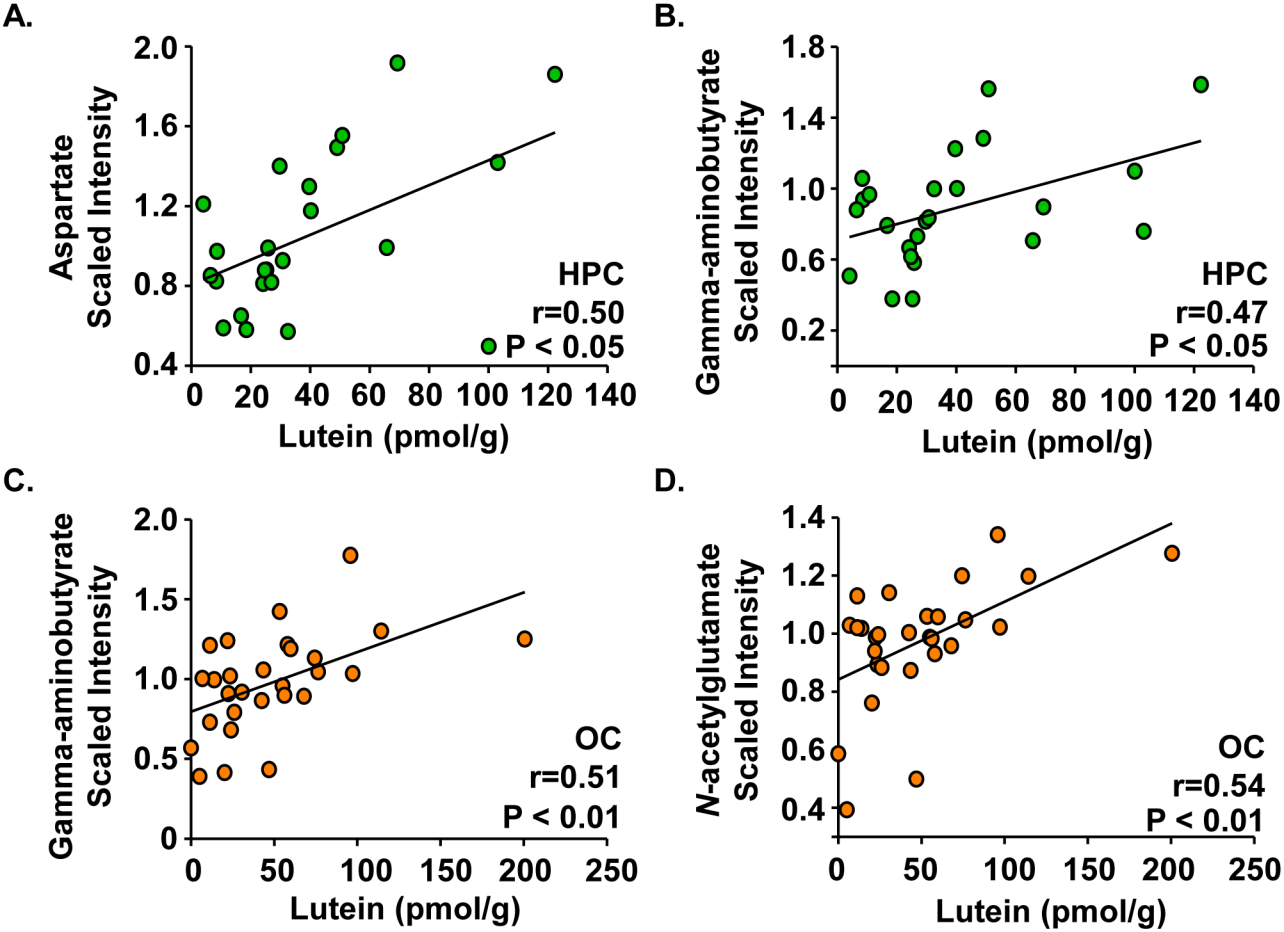
Amino acid neurotransmitters are positively correlated with lutein concentrations in infant hippocampus and occipital cortex. Post-mortem infant brain tissues (age 1 to 488 days) from the frontal cortex, hippocampus, and occipital cortex were analyzed for both lutein and amino acid neurotransmitters. Results are shown as metabolite (scaled intensity) by lutein concentration (pmol/g). In the hippocampus (n = 24), (A) aspartate and (B) gamma-aminobutyrate had moderate, positive correlations with lutein. In the occipital cortex (n = 28), (C) gamma-aminobutyrate, and (D) *N*-acetylglutamate had moderate, positive correlations with lutein. No moderate correlations were observed in the frontal cortex. Neurotransmitter:lutein correlations with r values ≥ |0.45| and P < 0.05 are reported. HPC: hippocampus; OC: occipital cortex.

Hippocampal lutein concentrations positively correlated with two amino acid neurotransmitters: aspartate (Fig 6A, r = 0.50, P < 0.05) and γ-aminobutyrate (GABA) (Fig 6B, r = 0.47, P < 0.05). Occipital cortex lutein concentrations positively correlated with GABA (Fig 6C, r = 0.51, P < 0.01) and *N*-acetylglutamate (Fig 6D, r = 0.54, P < 0.01).

### Homocarnosine is positively correlated with lutein concentrations in infant frontal cortex and hippocampus

**Fig 7:**
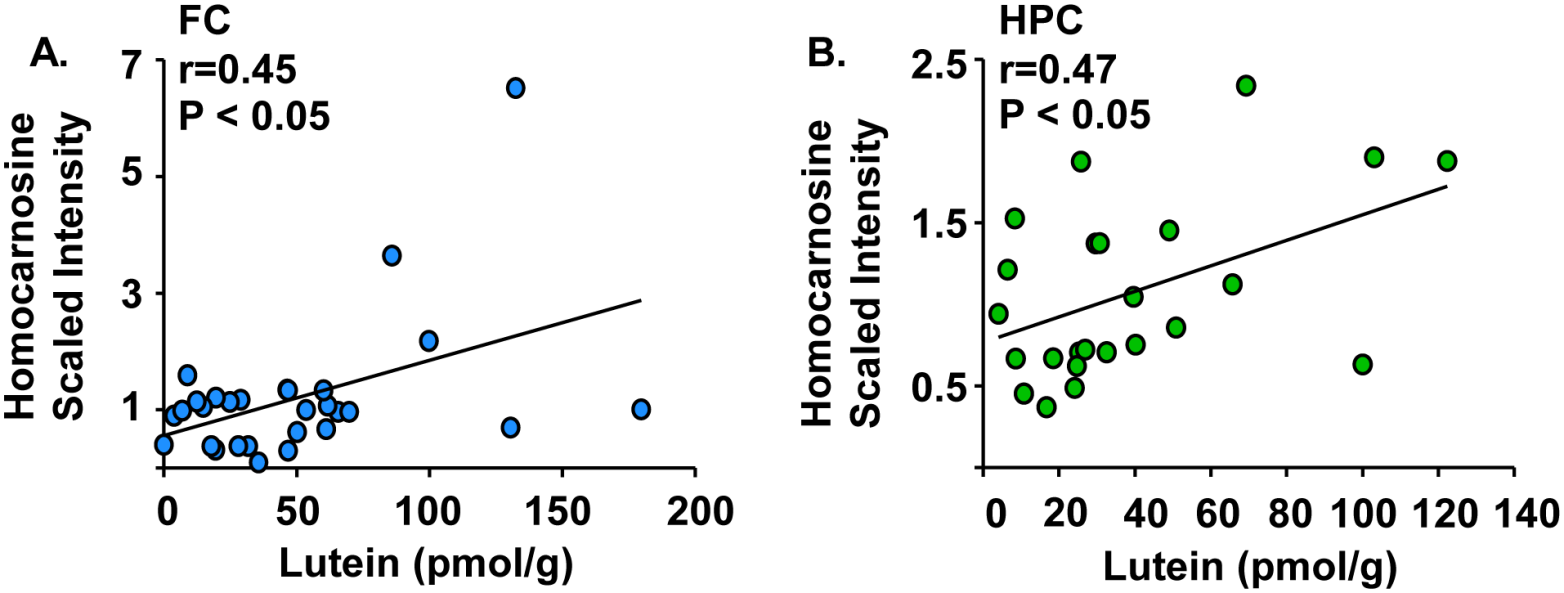
Homocarnosine is positively correlated with lutein concentrations in infant frontal cortex and hippocampus. Post-mortem infant brain tissues (age 1 to 488 days) from the frontal cortex, hippocampus, and occipital cortex were analyzed for both lutein and homocarnosine. Results are shown as homocarnosine (scaled intensity) by lutein concentration (pmol/g). Positive correlations between homocarnosine and lutein were observed in the (A) frontal cortex (n = 29) and (B) hippocampus (n = 24), but not the occipital cortex. Homocarnosine:lutein correlations with r values ≥ |0.45| and P < 0.05 are reported. FC: frontal cortex; HPC: hippocampus.

Lutein concentrations were analyzed for its relation to homocarnosine, an antioxidant exclusive to the central nervous system [30–32]. Analyses revealed that lutein concentrations positively correlated with increasing levels of homocarnosine in the frontal cortex (Fig 7A, r = 0.45, P < 0.05) and the hippocampus (Fig 7B, r = 0.47, P < 0.05).

## Discussion

Recently, lutein concentrations in serum, brain, and macular pigment were correlated with cognitive ability of older adults [10–14]. In addition, a recent study established that lutein is the predominant carotenoid in infant brain despite its limited presence in the diets of most infants [17]. Furthermore, this study also showed that lutein and its isomer zeaxanthin were the only carotenoids present in all infant brain regions studied. However, the mechanisms of action underlying a possible role for lutein in neurodevelopment have not yet been described. Therefore, we were interested in identifying candidate metabolite pathways through which lutein might influence neurodevelopment in the rapidly developing infant brain in the hopes of stimulating future mechanistic research. Toward this end, we coupled metabolomic analyses with known lutein concentrations in human infant brain samples from the frontal cortex, hippocampus, and occipital cortex. Our results indicate that lutein concentrations in these brain regions are correlated with a number of metabolites in a brain region specific manner.

Lutein was previously reported to be the dominant carotenoid in these brain tissues [17]. We report here that lutein concentrations did not change with age across these three brain regions. Therefore, as brain volume increased, lutein accretion did not exceed tissue expansion. These data support the hypothesis that lutein accretes at a steady rate to maintain a constant level of lutein in the brain during development despite brain volume growth or changes in lipid composition. An alternative explanation is that variation in dietary intake is the predominant determinate of brain lutein concentrations. In either case, a positive correlation between metabolites and lutein is unlikely to be a reflection of an increase in both with age. In addition, neither lutein nor any of the biochemical relationships described in this report correlated with post-mortem interval, supporting that these lutein relationships were not an artifact of biochemical degradation associated with time until tissue collection after death.

An important finding was that lutein concentrations were correlated with a number of fatty acids and lysophospholipids. These results are broadly consistent with previous reports that demonstrate lutein accumulates within areas of cell membranes rich in unsaturated phospholipids [33, 34]. In addition, previous reports established that carotenoids can modify the chemical and physical properties of cell membrane interactions with lipids thus resulting in altered membrane permeability and stability [35–38]. Our finding that lutein positively correlated with a number of lysophospholipids is consistent with these observations as these metabolites are intermediates of the lipid synthesis and remodeling pathways. Of perhaps even greater interest is the role of lysophospholipids as signaling molecules [39, 40]. Growing evidence supports that lysophospholipids are important bioactive compounds for intracellular and cell-cell signaling in the central nervous system. In particular, lysophosphatidic acid (LPA) induces cortical development and folding [41] and supports the later stages of oligodendrocyte maturation [42]. While the literature is growing for both LPA and sphingosine 1-phosphate, another well-studied lysophospholipid, relatively few lysophospholipid metabolites have been extensively explored to date. Therefore, the data from this report may reveal new research targets for future cell signaling studies.

Several findings from the present study suggest that lutein concentrations may be related to brain volume regulation during growth and development. The developing human brain experiences a period of rapid growth, beginning approximately midgestation and continuing through the first few years after birth, leading to a large increase in brain volume including a large expansion in lipid content [43]. In addition, generation of myelin, the lipid-dominant insulation of axons to increase the efficiency of signal transduction between neurons, begins after birth and continues for several years afterwards in humans [44, 45]. We found that lutein concentrations positively correlated with 1-octadecanol, a fatty alcohol. Long-chain fatty alcohols can act as substrates to support the production of myelin through the synthesis of plasmogens [46]. In addition to 1-octadecanol, both phosphate and NADH, metabolites associated with energy pathways, correlated with increased levels of lutein in the occipital cortex. This is consistent with the notion that lutein supports myelination during development as oligodendrocytes require extremely high metabolic rates during peak myelination [47]. Taken together, these results support the hypothesis that increased concentrations of these metabolites in combination with lutein may reveal an importance for this carotenoid in regulation of brain volume or brain structural growth, including myelin formation.

Lutein concentrations also positively correlated with *scyllo*-inositiol throughout the pediatric brain. This is noteworthy as inositols are sugar alcohols that can function as osmolytes and may be important for brain volume homeostasis in conjunction with other neuronal osmolytes [29]. Interestingly, analyses revealed that lutein concentrations negatively correlated with taurine in the hippocampus. Because taurine is also thought to be an important osmolyte in brain cells [25], this metabolite may be regulated to balance increases in other osmolytes such as *scyllo*-inositol. Alternatively, taurine is important during development for the visual cortex [48–50] and acts as a neurotransmitter through GABA and glycine receptors in brain [26–28]. Therefore, regulation of this metabolite in the hippocampus during development may be related to neurotransmission activity. In other studies, taurine was implicated as an antioxidant involved in membrane stabilization [51, 52]. Given that lutein is localized in membranes, it is possible that the inverse relationship is a reflection of protection of oxidizable lutein at the expense of taurine. Another hypothesis is that lutein and taurine could play coordinated roles in membrane stability or configuration. Certainly, the relationship between lutein and taurine in hippocampus warrants further investigation in the future.

A novel finding was that lutein concentration positively correlated with the amino acid neurotransmitters GABA (hippocampus and occipital cortex) and aspartate (hippocampus). GABA is thought to modulate neuronal proliferation and maturation, neurite outgrowth, and synapse formation [53–55], and aspartate is one of the major excitatory neurotransmitters in the brain [56]. Our results are consistent with recent findings showing that lutein intake was associated with improved cognitive function in the elderly [10, 11], and supplementation with lutein increased temporal processing speed in young, healthy adults [57]. These observations support the hypothesis that lutein may play a biochemical role in the development or remodeling of neurons. Lutein was also previously shown to protect neurons against oxidative stress [58, 59] indicating that lutein may aid neuronal activity through its antioxidant activity. However, it is also possible that lutein acts in a direct capacity to support neurotransmission. Of note, lutein concentration in the occipital cortex was positively correlated with *N*-acetylglutamate, but not glutamate. *N*-acetylglutamate is enzymatically generated from glutamate, an excitatory neurotransmitter. It is not surprising that the substrate and product for *N*-acetylglutamate synthase do not both correlate with lutein as regulation of enzyme activity is complex and these compounds exhibit different roles. Taken together, our data suggest that lutein may be important for neuronal activity during development.

Lutein is best known for its roles as an antioxidant and a UV filter to protect the macula. The most widely recognized activity of lutein is its role as an antioxidant particularly in the protection of the photoreceptor cell layer from oxidative damage [3–5]. The brain is especially vulnerable to oxidative stress during development [60]. Therefore, it is of interest to note that lutein may support antioxidant activity in the infant brain. Lutein concentrations positively correlated with homocarnosine in the frontal cortex and hippocampus. Homocarnosine is found in high concentrations in the brain and is implicated as a neuroprotective antioxidant [30, 61].

This study is the first known metabolic report on the infant brain metabolome, and certainly the first to relate the metabolome to lutein. Here, we report that lutein in the human infant brain correlated with lipid pathway metabolites, energy pathway metabolites, brain osmolytes, amino acid neurotransmitters, and the antioxidant homocarnosine. These data reveal a number of metabolites that are candidates in describing the functional importance for lutein on brain development and cognition. It should be noted that there could be an interaction between general nutrition and these correlations and that the concentrations of lutein in brain tissue may be serving as a marker of overall nutritional status. However, in our previous publications we observed that among the carotenoids, lutein is preferentially taken up into human brain tissue [17, 62] and is the most consistently related to cognitive function [10], supporting a possible unique role in cognition. While we are aware that metabolomic correlations do not provide definitive proof for how lutein could impact neurodevelopment, these exploratory analyses provide clues to how lutein may influence learning and memory. Identifying potential pathways for lutein activity will hopefully aid future biochemical studies in illuminating the mechanisms by which lutein supports cognition in infants as well as adults. In addition, future studies may reveal the importance of optimal lutein intake during development as inadequate accretion of lutein in infant brains may impact brain maturation.

## Supporting Information

S1 Dataset All Metabolite:Lutein Correlations. An exploratory study relating metabolite signatures and lutein was completed on post-mortem infant brain tissues (age 1 to 488 days) from the frontal cortex, hippocampus, and occipital cortex. A total of 1276 correlations were carried out across all brain regions. Five metabolites fell below the detection limit in the frontal cortex, and therefore, correlations were not carried out for these (“not detected”). Of 427 metabolites analyzed, 257 were metabolites of known identity. Correlations from all known 257 metabolites are reported.
(XLSX)

S2 Dataset Post-Mortem Interval Correlations. An exploratory study relating metabolite signatures and lutein was completed on post-mortem infant brain tissues (age 1 to 488 days) from the frontal cortex, hippocampus, and occipital cortex. Samples were then analyzed for correlations between on all 257 known metabolites and post-mortem interval, or the time between death and tissue collection.
(XLSX)

S1 Fig Lutein:Sex Correlations. Post-mortem infant brain tissues (age 1 to 488 days) were analyzed for lutein by HPLC. Increasing lutein concentration (pmol/g) did not correlate with sex in the (A) frontal cortex (n = 29), (B) hippocampus (n = 24), or (C) occipital cortex (n = 28). FC: frontal cortex; HPC: hippocampus; OC: occipital cortex; n.s.: not-significant.
(PDF)

S2 Fig Lutein:Post-Mortem Interval Correlations. Post-mortem infant brain tissues (age 1 to 488 days) were analyzed for lutein by HPLC. Increasing lutein concentration (pmol/g) did not correlate with the post-mortem interval, the time between death and tissue collection, in the (A) frontal cortex (n = 29), (B) hippocampus (n = 24), or (C) occipital cortex (n = 28). FC: frontal cortex; HPC: hippocampus; OC: occipital cortex; n.s.: not-significant.
(PDF)
