# Supplementary figures and images for: Exploratory Metabolomic Analyses Reveal Compounds Correlated with Lutein Concentration in Frontal Cortex, Hippocampus, and Occipital Cortex of Human Infant Brain
Source: PLoS One. 2015 Aug 28;10(8):e0136904. doi: 10.1371/journal.pone.0136904 (PMC4552625; doi:10.1371/journal.pone.0136904)

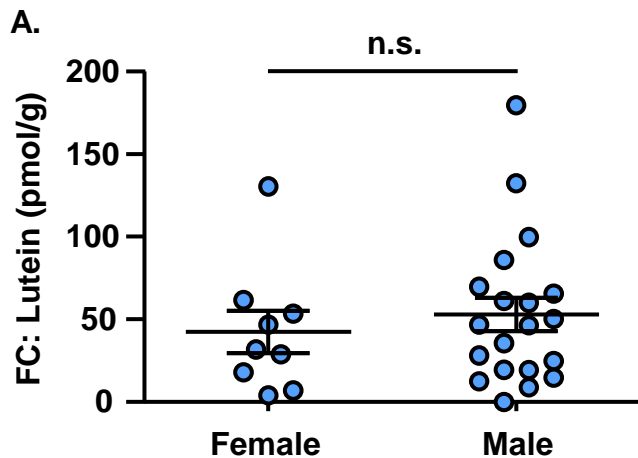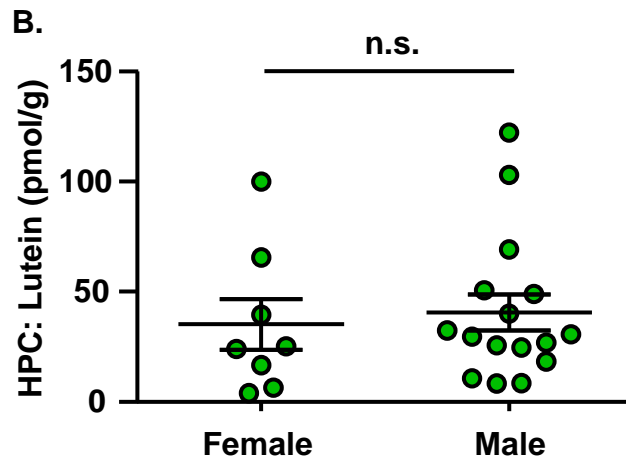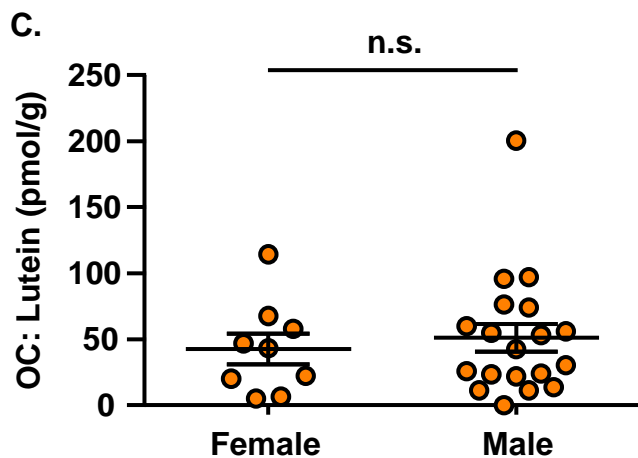

Supplement: S1 Fig — Post-mortem infant brain tissues (age 1 to 488 days) were analyzed for lutein by HPLC. Increasing lutein concentration (pmol/g) did not correlate with sex in the (A) frontal cortex (n = 29), (B) hippocampus (n = 24), or (C) occipital cortex (n = 28). FC: frontal cortex; HPC: hippocampus; OC: occipital cortex; n.s.: not-significant. (PDF) [file pone.0136904.s003.pdf]

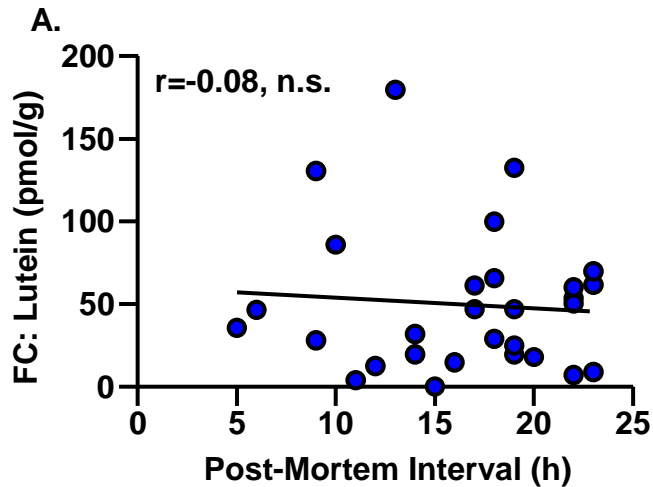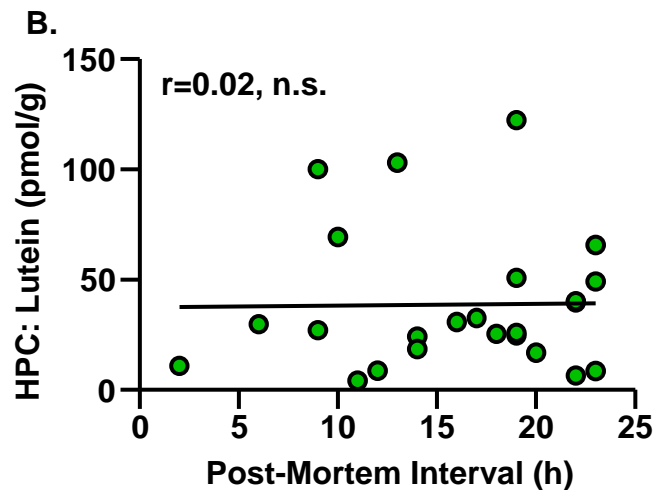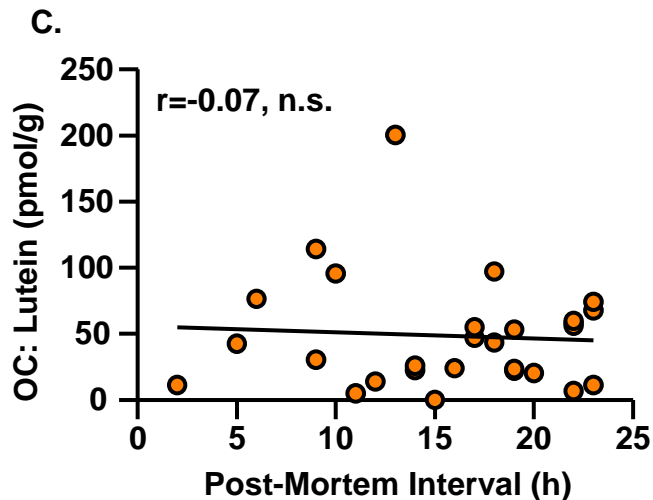

Supplement: S2 Fig — Post-mortem infant brain tissues (age 1 to 488 days) were analyzed for lutein by HPLC. Increasing lutein concentration (pmol/g) did not correlate with the post-mortem interval, the time between death and tissue collection, in the (A) frontal cortex (n = 29), (B) hippocampus (n = 24), or (C) occipital cortex (n = 28). FC: frontal cortex; HPC: hippocampus; OC: occipital cortex; n.s.: not-significant. (PDF) [file pone.0136904.s004.pdf]
